# Preparation of a Crosslinked Poly(imide-siloxane) for Application to Transistor Insulation

**DOI:** 10.3390/polym14245392

**Published:** 2022-12-09

**Authors:** Hyeong-Joo Park, Ju-Young Choi, Seung-Won Jin, Seung-Hyun Lee, Yun-Je Choi, Dam-Bi Kim, Chan-Moon Chung

**Affiliations:** Department of Chemistry, Yonsei University, Wonju 26493, Republic of Korea

**Keywords:** IGBT, insulating material, poly(imide-siloxane), crosslinking, thermal property

## Abstract

Insulated gate bipolar transistor (IGBT) is an important power device for the conversion, control, and transmission of semiconductor power, and is used in various industrial fields. The IGBT module currently uses silicone gel as an insulating layer. Since higher power density and more severe temperature applications have become the trend according to the development of electronic device industry, insulating materials with improved heat resistance and insulation performances should be developed. In this study, we intended to synthesize a new insulating material with enhanced thermal stability and reduced thermal conductivity. Poly(imide-siloxane) (PIS) was prepared and crosslinked through a hydrosilylation reaction to obtain a semi-solid Crosslinked PIS. Thermal decomposition temperature, thermal conductivity, optical transparency, dielectric constant, and rheological property of the Crosslinked PIS were investigated and compared to those of a commercial silicone gel. The Crosslinked PIS showed high thermal stability and low thermal conductivity, along with other desirable properties, and so could be useful as an IGBT-insulating material.

## 1. Introduction

The insulated gate bipolar transistor (IGBT) is a device for the conversion, control, and transmission of semiconductor power, and is used in various industrial fields such as automobiles, aviation, and home appliances [1,2,3]. According to the development of the electronic device industry, the device requirements of size, weight, reliability, and durability are becoming stricter. It is especially expected that future IGBT devices would undergo a temperature increase to 200 °C during their operation. More efficient and durable insulating materials are required since higher power density and more severe temperature applications have become the trend [4,5].

Silicone gels are generally used for insulating high voltage multichip power assemblies for their high softness and insulating properties [1,5]. The gels have Si-O-Si groups with a bond angle between 104° and 180°, introducing flexibility to the crosslinked polymer chains [6,7,8]. The silicone gel fills the IGBT module in order to protect it and ensure sufficient electrical and thermal insulation (Figure S1 in Supplementary Materials) [1,2,3,9]. However, it is considered that the silicone gel has limited stability at the increased operating temperatures of the future IGBT module. Therefore, in order to improve the thermal stability of the silicone polymer and prevent heat dissipation from the IGBT module, insulating materials with improved heat resistance and low thermal conductivity should be developed [1,2,3].

Polyimides have advantageous properties such as good mechanical properties, thermal stability, and chemical resistance [10,11,12,13,14,15,16,17]. Polyimides are also known to have low dielectric constants and thermal conductivity, so they have been widely used in microelectronics, automobiles, and aerospace industries as electronic packaging and electrical insulating materials [18,19,20]. On the other hand, poly(imide-siloxane)s (PISs) have been synthesized using amine-terminated siloxane compounds [21,22,23,24,25,26,27,28]. By introducing the siloxane backbone into the polyimides, the PISs have some beneficial properties such as enhanced solubility, low temperature flexibility, thermal and UV stability, adhesive properties, oxidative resistance, and proton conductivity. Due to these properties, PISs have been studied for various applications including fuel cell proton exchange membranes [21], gas permeation membranes [22], microelectronic-insulating layers [23,24], atomic oxygen erosion-resistant materials [25], and adhesives [26,27]. In addition, a PIS composite containing a high amount of alumina has been prepared for application as a heat-dissipating material [28]. However, PISs have not been studied as an insulating material for the IGBT module. For applications to IGBT-insulating materials, high thermal stability, low thermal conductivity, low dielectric constant, high optical transparency, and appropriate viscoelastic properties are required [1,29].

In this work, we first developed a new PIS gel for application as an insulating material for IGBT. A PIS was prepared and crosslinked through a hydrosilylation reaction to obtain a semi-solid Crosslinked PIS. Thermal stability, thermal conductivity, optical transparency, dielectric constant, and rheological property of the Crosslinked PIS were investigated and compared to those of the commercial silicone gel.

## 2. Materials and Methods

### 2.1. Materials

4,4′-(Hexafluoroisopropylidene)diphthalic anhydride (6FDA), allyl isocyanate, and platinum(0)-1,3-divinyl-1,1,3,3-tetramethyldisiloxane complex solution (in xylene) were purchased from Sigma-Aldrich Korea (Seoul, Republic of Korea). Bis(3-aminopropyl)-terminated polydimethylsiloxane (PDMS) ($\bar{M}$_n_ ~3000) and trimethylsiloxy-terminated methylhydrosiloxane-dimethylsiloxane (MHS-PDMS) copolymers (MHS unit mole % = 0.5–1.5) were purchased from Gelest (Morrisville, PA, USA). Tetrahydrofuran (THF) and methanol were purchased from Samchun Chemicals (Seoul, Republic of Korea), distilled from N_2_/benzophenone, and stored under nitrogen atmosphere. Pyridine was purchased from Alfa Aesar Chemicals (Ward Hill, MA, USA). Acetic anhydride was purchased from Junsei Chemical Co., Ltd. (Tokyo, Japan). A commercial IGBT module was purchased from I.G. International (Busan, Republic of Korea) and its insulating material was characterized. It is considered that the insulating material is a PDMS-based gel (i.e., silicone gel) because its IR spectrum is practically the same as that of an authentic PDMS (Figure S2).

### 2.2. Instruments

The drying and thermal treatment of samples were conducted with a vacuum oven (LO-VS330N, LKLAB KOREA Inc., Namyangju, Republic of Korea). Fourier Transform Infrared (FT-IR) spectroscopy was carried out using a Spectrum One B FT-IR spectrometer (PerkinElmer, Inc., Waltham, MA, USA) using the KBr pellet technique with the following scan parameters: scan range 400–4000 cm^−1^; number of scan 1; resolution 4 cm^−1^. Proton nuclear magnetic resonance (^1^H NMR) spectra of samples dissolved in CDCl_3_ were acquired using a Bruker Avance II 400 MHz spectrometer (Bruker Corporation, Billerica, MA, USA). Gel permeation chromatography (GPC) was carried out using a chromatographic system (Thermo Scientific, Waltham, MA, USA) in refractive index mode. The GPC was conducted using a doubly connected Showa Denko Shodex KF-806L column at 40 °C and THF as an eluent at a flow rate of 1.0 mL/min, and the results were calibrated with respect to polystyrene standards. In the GPC analysis, the number average molecular weight ($\bar{M}$_n_), weight average molecular weight ($\bar{M}$_w_), and polydispersity index (PDI) were measured. Thermal analyses were carried out under a nitrogen atmosphere with a balance flow rate of 40 mL/min and a furnace flow rate of 60 mL/min using a Discovery TGA 55 (TA instrument, Inc., New Castle, DE, USA) at a heating rate of 10 °C/min. Thermal conductivities (in-plane) of the samples were measured using a LFA 467 Nanoflash (NETZSCH Korea Co., Ltd., Koyang, Republic of Korea) at 25 °C under a nitrogen atmosphere. Elemental analysis was carried out using a Flash 2000 (Thermo Fisher Scientific, Waltham, MA, USA). Ultraviolet–visible spectroscopy was carried out using a Lamda 25 UV/Vis spectrometer (PerkinElmer, Inc., Waltham, MA, USA). Dielectric spectroscopy was performed using a E4980A dielectric spectrometer (KEYSIGHT, San Diego, CA, USA) at 22 °C in the frequency range of 50–1,000,000 Hz. The rheological property was studied using an Advanced Rheometric Expansion System (ARES, Rheometric Scientific, New Castle, DE, USA).

### 2.3. Preparation of PAAS Solution

**Scheme 1:**
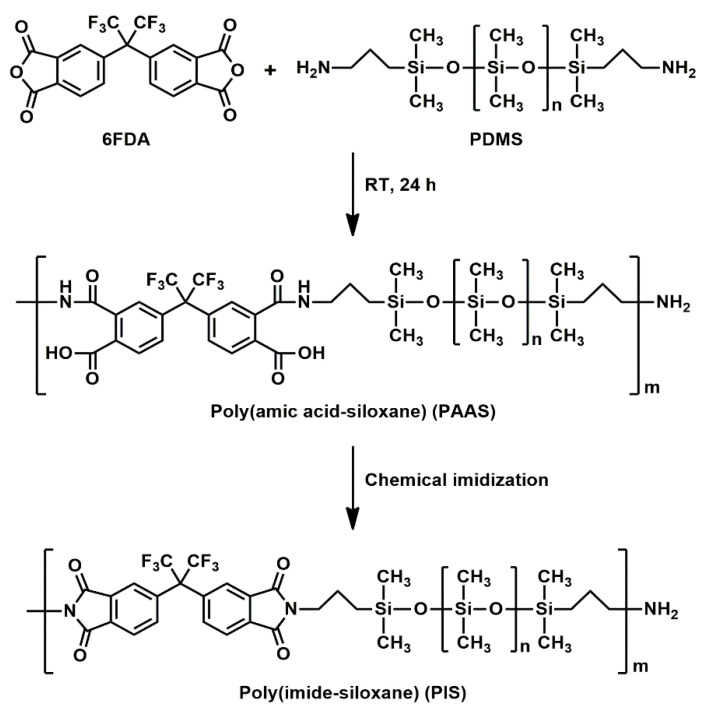
Synthesis of PIS.

Poly(amic acid-siloxane) (PAAS) was prepared using a molar ratio of 6FDA:PDMS of 1:1.05 (Scheme 1). Firstly, a dried 500 mL flask was charged with PDMS (31.5 g, 0.0105 mol) in THF (202 mL) under a nitrogen atmosphere, and the solution was stirred at room temperature for 1 h. After 6FDA (4.44 g, 0.0100 mol) in THF (10 mL) was added, and the resultant solution was stirred at room temperature for 24 h to yield a PAAS solution. PAAS: FT-IR (KBr): 1724 cm^−1^ (C=O stretching of carboxyl), 1640 cm^−1^ (C=O stretching of amide), 1260 cm^−1^ (symmetric deformation of -CH_3_ group in -Si(CH_3_)_2_-), 1092 and 1021 cm^−1^ (Si-O-Si stretching), 802 cm^−1^ (Si-C stretching).

### 2.4. Preparation of PIS

The preparation of PIS is illustrated in Scheme 1. After the synthesis of PAAS, pyridine (1.6 mL) and acetic anhydride (1.8 mL) were added to the PAAS solution. The mixture was refluxed (70 °C) under stirring for 6 h under a nitrogen atmosphere. After the reaction, the mixture was cooled to room temperature and poured into 250 mL of water. A yellow viscous product was collected, washed thoroughly with water and methanol, and dried at 50 °C under vacuum overnight to give a viscous PIS. PIS: FT-IR (KBr): 1779 cm^−1^ (imide C=O asymmetric stretching), 1726 cm^−1^ (imide C=O symmetric stretching), 1363 cm^−1^ (imide C-N stretching), 1260 cm^−1^ (symmetric deformation of -CH_3_ group in -Si(CH_3_)_2_-), 1092 and 1021 cm^−1^ (Si-O-Si stretching), 802 cm^−1^ (Si-C stretching). ^1^H NMR (400 MHz, CDCl_3_) δ_H_: 0.05 [s, Si-CH_3_], 0.53–0.58 [t, Si-CH_2_], 1.68–1.70 [m, CH_2_-CH_2_-CH_2_], 3.64–3.68 [t, N-CH_2_], 7.74–7.89 [m, aromatic H]. Anal. Calcd: C, 36.34; H, 7.48; N, 0.82. Found: C, 36.31; H, 7.68; N, 0.86.

### 2.5. Preparation of Allyl-PIS

**Scheme 2:**
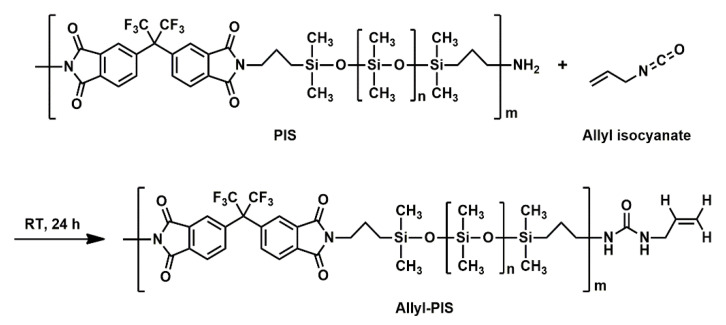
Synthesis of Allyl-PIS.

The preparation of Allyl-PIS is illustrated in Scheme 2. The PIS (30 g) obtained above was dissolved in THF (100 mL), and allyl isocyanate (2.8 mL) was added. The mixture was stirred at room temperature for 24 h under a nitrogen atmosphere. During the reaction, the amino end group of the PIS reacted with allyl isocyanate to form urea linkage. After the reaction, the mixture was poured into 250 mL of water. The yellow viscous product was collected, washed thoroughly with water and methanol, and dried at 50 °C under vacuum overnight to give Allyl-PIS. Allyl-PIS: ^1^H NMR (400 MHz, CDCl_3_) δ_H_: 0.05 [s, Si-CH_3_], 0.53–0.58 [t, Si-CH_2_], 1.64–1.72 [m, CH_2_-CH_2_-CH_2_], 3.64–3.68 [t, N-CH_2_], 4.28–4.29 [d, CH_2_ of allyl group], 5.18–5.27 [m, CH_2_ of allyl group], 5.81–5.89 [m, CH of allyl group], 7.74–7.89 [m, aromatic H].

### 2.6. Preparation of Crosslinked PIS

**Scheme 3:**
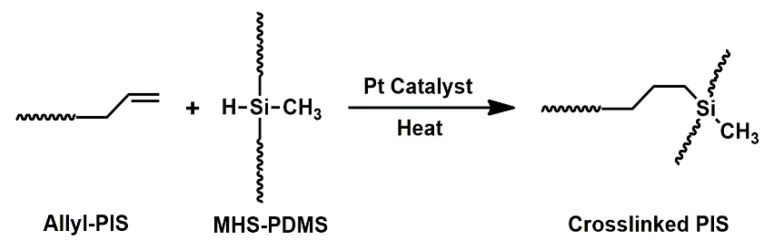
Synthesis of Crosslinked PIS.

The crosslinking reaction of Allyl-PIS was performed to synthesize a Crosslinked PIS gel material (Scheme 3). The crosslinker (MHS-PDMS) and the catalyst platinum(0)-1,3-divinyl-1,1,3,3-tetramethyldisiloxane complex solution were added to Allyl-PIS and mixed thoroughly. The mixture was poured into a boat-shaped mold and degassed with an evaporator, and then put in a vacuum oven. The mixture was heated under vacuum to simultaneously perform degassing and the hydrosilylation reaction. Heating was conducted at 40 °C overnight, 70 °C, 100 °C and 120 °C for 1 h each, and finally at 140 °C for 3 h.

## 3. Results and Discussion

### 3.1. Preparation of PAAS and PIS

As illustrated in Scheme 1, PAAS was synthesized from 6FDA and PDMS with a molar feed ratio of 6FDA:PDMS of 1:1.05. A higher molar amount of PDMS than that of 6FDA was used so that the end groups of PIS would mainly be amino groups. PIS was obtained as a pale-yellow viscous liquid through the chemical imidization of PAAS. Table 1 lists the molecular weights and polydispersity index (PDI) of PIS.

**Figure 1:**
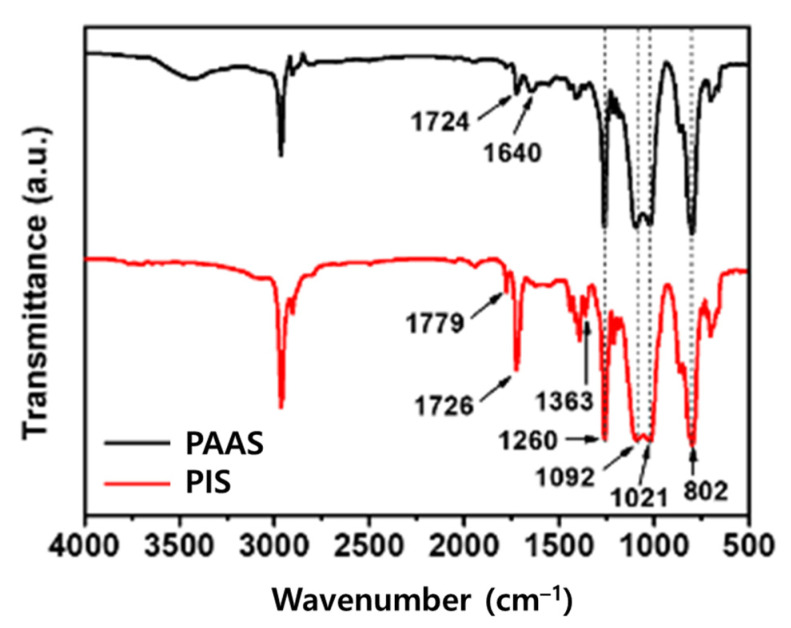
FT-IR spectra of PAAS and PIS.

As shown in Figure 1, the FT-IR spectrum of PAAS showed absorption bands at 1724 cm^−1^ (carboxyl) and 1640 cm^−1^ (amide), owing to C=O stretching. The absorption bands at 1021 and 1092 cm^−1^ are due to the stretching vibration of the Si-O-Si group in the PDMS structure, and the absorption band at 1260 cm^−1^ is attributed to the symmetrical deformation of the -CH_3_ group in the -Si(CH_3_)_2_- group. The absorption band at 802 cm^−1^ corresponds to Si-C vibration [30,31,32,33,34]. In the spectrum of PIS, absorption bands at 1779 cm^−1^ due to imide C=O asymmetric stretching, at 1726 cm^−1^ due to imide C=O symmetrical stretching, and at 1363 cm^−1^ due to C–N stretching were observed. The absorption bands of the PDMS structure described above were also observed in the PIS spectrum.

**Figure 2:**
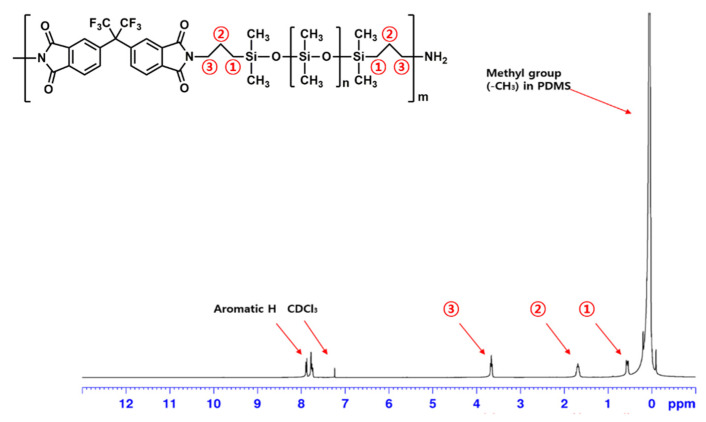
^1^H NMR spectrum of PIS.

The structure of PIS was also confirmed by ^1^H NMR spectroscopy and elemental analysis. In the NMR spectrum of PIS (Figure 2), the peak of the methyl group (-CH_3_) in the PDMS unit was identified (0.05 ppm). In addition, three methylene group (-CH_2_-) peaks at both ends of the PDMS were identified (➀ 0.53–0.58, ➁ 1.68–1.70, and ➂ 3.64–3.68 ppm) and proton peaks in the aromatic ring were observed at 7.74–7.89 ppm. In the elemental analysis of PIS, the calculated values of C, H, and N were 36.34%, 7.48%, and 0.82%, respectively, and the measured values were 36.31%, 7.68%, and 0.86%, respectively, indicating good agreement between the calculated and measured values.

### 3.2. Preparation of Allyl-PIS and Crosslinked PIS

**Figure 3:**
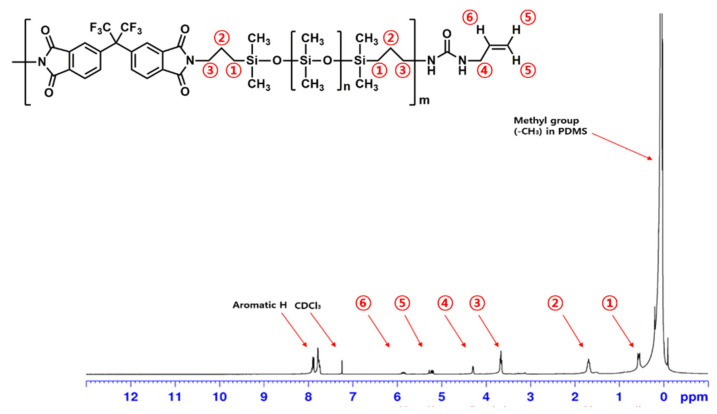
^1^H NMR spectrum of Allyl-PIS.

The preparation of Allyl-PIS is illustrated in Scheme 2. The Allyl group was introduced into the PIS end group by reacting allyl isocyanate with the amino end group of PIS, and was used for the crosslinking of PIS later. Allyl-PIS was obtained as a viscous liquid and identified by FT-IR and ^1^H NMR spectroscopy. The FT-IR spectrum of Allyl-PIS was practically the same as that of PIS (Figure S3). In the ^1^H NMR spectrum of Allyl-PIS (Figure 3), the peak of the methyl group (-CH_3_) in the PDMS unit was identified (0.05 ppm). In addition, three methylene group (-CH_2_-) peaks at both ends of the PDMS were identified (➀ 0.53–0.58, ➁ 1.64–1.72, ➂ 3.64–3.68 ppm). Allyl proton peaks were identified (➃ 4.28–4.29, ➄ 5.18–5.27, and ➅ 5.81–5.89 ppm) and proton peaks in the aromatic ring were identified (7.74–7.89 ppm).

The synthesized Allyl-PIS was crosslinked through the hydrosilylation reaction between Si-H and allyl groups to obtain Crosslinked PIS as a semi-solid product. Allyl-PIS was reacted in the presence of the crosslinker (MHS-PDMS) and catalyst (platinum(0)-1,3-divinyl-1,1,3,3-tetramethyldisiloxane complex solution) using reaction mixtures with various compositions. During the crosslinking reactions, the reaction mixtures showed a tendency to foam, probably due to the evaporation of residual solvents. Foaming may cause the nonuniformity of properties, decrease in mechanical properties, and optical opacity. On the other hand, since the PIS rapidly converted to a dark brown substance above 150 °C, the reaction was performed at 140 °C or below. However, when the reaction mixtures were directly heated up to 140 °C, foaming inside the products occurred. In order to avoid foaming, the reaction was conducted by stepwise heating under vacuum. The reaction was first performed at 40 °C overnight and then at 70 °C, 100 °C and 120 °C for 1 h each, and finally at 140 °C for 3 h. The composition of the reaction mixtures and state of the products are summarized in Table 2.

We attempted to find the optimum reaction composition to give non-foamed semi-solid (Table 2). The softness and adhesive properties of semi-solid such as silicone gel make it a good choice to avoid the defects from impurities and thermomechanical stresses [35]. First, the concentration of the catalyst was fixed at 0.5 or 1 wt% and the crosslinking reactions were performed by varying the concentration of the crosslinker (1~7 wt%). When the crosslinker concentration was below 3 wt%, enough crosslinking density could not be obtained, and the products remained as viscous liquids (Run No. 1 and 5). In contrast, when the crosslinker concentration was higher than 3 wt%, the crosslinking reaction rate was so high that the internal gas was trapped inside the product (Run No. 3, 4, 7 and 8). Through this, the optimum concentration of the crosslinker was determined to be 3 wt% (Run No. 2 and 6).

Next, the concentration of the crosslinker was fixed at 3 wt%, and then the crosslinking reactions were performed by varying the concentration of the catalyst (0.1 or 2 wt%) (Run No. 9 and 10 in Table 2). When the concentration of the catalyst was 0.1 wt%, enough crosslinking density could not be obtained, and the product remained as a viscous liquid (Run No. 9). In contrast, when the catalyst concentration was 2 wt%, the crosslinking reaction rate was so high that foaming occurred (Run No. 10). Considering the commercial aspect, the lower concentration of catalyst (0.5 wt%) should be more advantageous. Finally, the optimum concentration of the crosslinker and catalyst was determined to be 3 and 0.5 wt%, respectively (Run No. 2). The thermal, optical, electrical, and rheological properties of the product from Run No. 2 were investigated and the results are described below.

**Figure 4:**
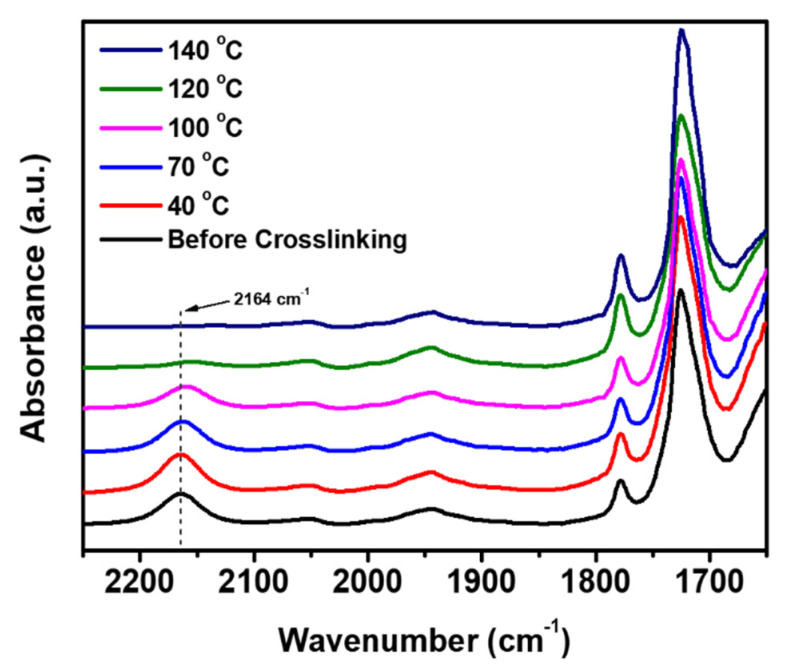
FT-IR spectra showing the progress of the crosslinking reaction under stepwise heating.

**Figure 5:**
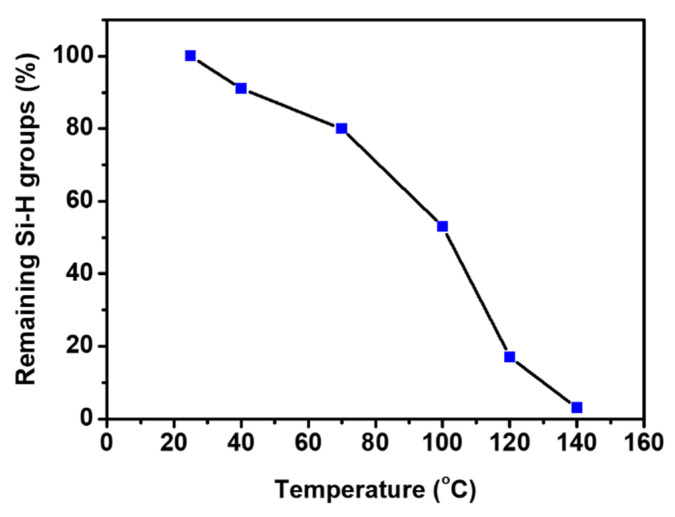
Remaining Si-H groups of the progress of the crosslinking reaction.

The progress of the crosslinking reaction of the mixture having the Run No. 2 composition was investigated by FT-IR spectroscopy (Figure 4). It was expected that the absorbance of Si-H band (2164 cm^−1^) would reduce as the reaction between Si-H and allyl groups proceeds [36]. As shown in Figure 4, the remaining Si-H groups decreased with the increasing reaction temperature. The absorbance value (at 2164 cm^−1^) of a mixture sample after each stepwise heating was compared with that of the sample before heating. The remaining percent of the Si-H groups was determined to be 91%, 80%, 53%, and 17% after stepwise heating at 40 °C, 70 °C, 100 °C, and 120 °C for 1 h each, respectively (Figure 5 and Table S1). The remaining percent of the Si-H groups decreased to 3% when the mixture was finally heated at 140 °C for 3 h after the previous stepwise heating. These results indicate that the crosslinking reaction was almost completed under the heating conditions. As described above, one drawback of our insulating material is that the crosslinking reaction mixtures show a tendency to foam during the crosslinking reaction. This drawback needs to be improved for the facile application of the new insulator.

### 3.3. Thermal Properties of Crosslinked PIS

**Figure 6:**
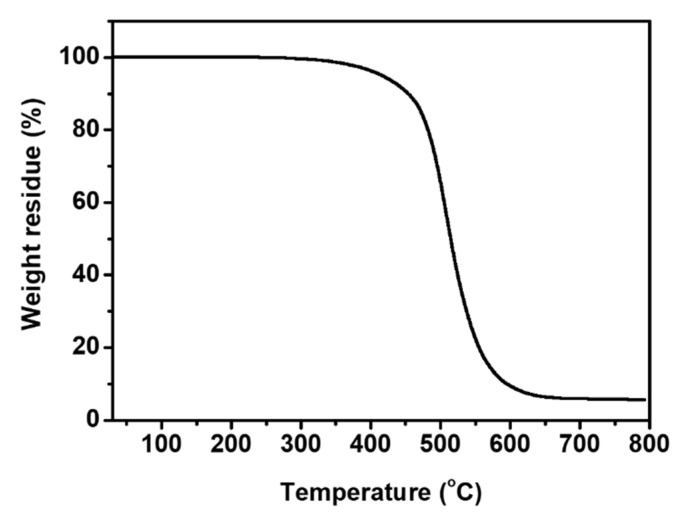
TGA curve of Crosslinked PIS.

Thermogravimetric analysis (TGA) was conducted to study thermal decomposition temperatures (T_5_ and T_10_) of the Crosslinked PIS (Figure 6 and Table 3). T_5_ and T_10_ values of the Crosslinked PIS were measured to be 416 °C and 454 °C, respectively. The decomposition temperatures of the Crosslinked PIS are higher than those of the commercial silicone gel (T_5_ = 378 °C, T_10_ = 422 °C) (Table S2 and Figure S4). This result is attributable to the incorporation of thermally stable imide moiety into the PDMS structure.

The thermal conductivity of a transistor insulating material is required to be low because the heat conduction between electronic devices should be minimal. Thermal conductivity (*k*) of the Crosslinked PIS and commercial gel was investigated by the laser flash method (LFA) at room temperature (25 °C). The *k* value can be calculated using the equation *k* = α × ρ × C_p_, where α is thermal diffusivity; ρ, measured density; and C_p_, specific heat capacity. The *k* value of the Crosslinked PIS was determined to be 0.11 W/m·K (Table 3), which is quite lower than that of the commercial gel (0.19 W/m·K) (Table S2). This is basically attributable to the imide structure that is known to have a much lower *k* value (polyimide, around 0.1 W/m·K) than PDMS (*k* = 0.25 W/m·K) [20]. It was considered that the incorporation of the 6FDA unit containing the bulky -CF_3_ groups leads to an increase in free volume, further lowering the thermal conductivity [37,38]. The Crosslinked PIS may be useful as an insulating layer of the transistor requiring high thermal stability and quite low thermal conductivity.

### 3.4. Optical Property of Crosslinked PIS

**Figure 7:**
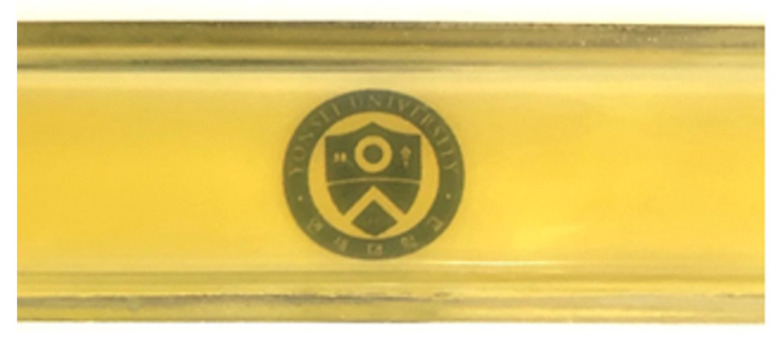
A photograph of Crosslinked PIS covering a printed design.

**Figure 8:**
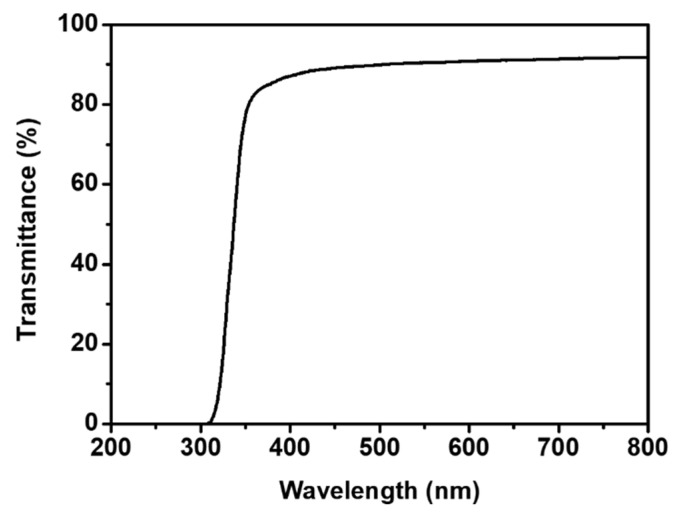
Transmittance of Crosslinked PIS.

IGBT modules may undergo partial discharge, and optical detection of the possible problem is sometimes carried out. Therefore, their insulating layer needs to be transparent [39,40]. A photograph of Crosslinked PIS covering a printed design is shown in Figure 7. While Crosslinked PIS had a yellow color, it is basically transparent. The optical transmittance of Crosslinked PIS was investigated by UV–Vis spectroscopy (Figure 8). For measurement, a Crosslinked PIS sample was coated on a quartz plate (coating thickness = 100 μm). Transmittance of the Crosslinked PIS at 550 nm was 91%, indicating its high optical transparency. Transmittance of the commercial IGBT gel was measured to be 89% (Figure S5), indicating that the transmittance of Crosslinked PIS is similar to that of commercial gel. The high transparency of the Crosslinked PIS is attributed to the presence of the siloxane segments that are difficult to form conjugated structures and intermolecular charge transfer complexes. The high transparency is partly attributable to bulky and electron-withdrawing CF_3_ groups in the PIS, which prohibits the formation of charge-transfer complexes by sterically repelling adjacent chains [41]. For comparison, pyromellitic dianhydride (PMDA)-4,4′-oxydianiline (ODA) PI, which is known to have a strong charge-transfer interaction, shows a much lower transmittance of 66% at 550 nm [42].

### 3.5. Electrical Property of Crosslinked PIS

Low dielectric constant is one of the important properties for insulating materials in electronic devices [43]. The dielectric constant of Crosslinked PIS and the silicone gel was measured to be 3.11 and 2.81, respectively. It is considered that the relatively low dielectric constant of the Crosslinked PIS is mainly due to the PDMS structure. The siloxane segment increases the free volume, effectively reducing the number of polarizing groups per unit volume [26]. While the polarizability of the Si-O bond in PDMS is higher than that of organic nonpolar polymers, the side methyl groups prevent Si-O dipoles from approaching each other too closely, thus, the dielectric permittivity of PDMS is very low [44]. On the other hand, it is known that the dielectric constant of polyimides can be lowered by the incorporation of fluorine atoms that increase hydrophobicity and decrease total polarizability [38]. Although the dielectric constant of Crosslinked PIS is slightly higher than that of silicone gel, the value is sufficiently low; therefore, it may be useful for insulation applications requiring a low dielectric constant [38,45].

### 3.6. Rheological Property of Crosslinked PIS

**Figure 9:**
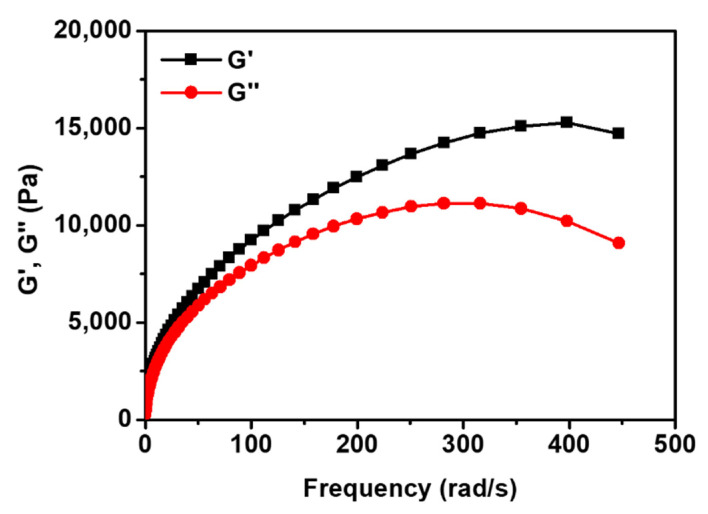
Storage modulus (G′) and loss modulus (G″) of Crosslinked PIS.

Viscoelastic measurements of Crosslinked PIS and the commercial silicone gel were carried out using a rheometer (Figure 9 and Figure S6). The frequency range varied from 0.1 to 500 rad/s and the strain was 2.0% at room temperature (25 °C). The value of storage modulus (G′) represents elasticity, and loss modulus (G″) represents viscosity. In the curve of the rheological property of the Crosslinked PIS (Figure 9), elasticity is more predominant than viscosity, indicating that the Crosslinked PIS is elastic rather than viscous. This tendency of the Crosslinked PIS is similar to that of the silicone gel (Figure S6). The Crosslinked PIS can be classified into a polymer gel because a gel is defined as a dilute crosslinked material, which exhibits no flow when in the steady state [46]. A gel form of the insulating material is advantageous because the gel can have self-healing capability [1].

## 4. Conclusions

PIS was synthesized from 6FDA and PDMS, and its end groups were modified using allyl isocyanate. The resultant Allyl-PIS was crosslinked by the hydrosilylation reaction in the presence of the crosslinker and Pt catalyst. The properties of the Crosslinked PIS were investigated and compared to those of the commercial silicone gel. T_5_ and T_10_ values of the Crosslinked PIS were 416 °C and 454 °C and those of the silicone gel were 378 °C and 422 °C, respectively, indicating higher thermal stability of Crosslinked PIS than the silicone gel. The thermal conductivity value of Crosslinked PIS was 0.11 W/m·K, which was much lower than that of the silicone gel (0.19 W/m·K). The higher thermal stability and lower thermal conductivity of the Crosslinked PIS could be achieved by incorporating the imide ring structure into a PDMS-based structure. Besides, the dielectric constant of the Crosslinked PIS was 3.11, which means that it is a low dielectric material. Crosslinked PIS showed a high optical transmittance of over 90% at 550 nm, allowing for the optical detection of a possible problem of the material. The elasticity of the crosslinked PIS was higher than the viscosity, which means that Crosslinked PIS is a polymer gel. Based on these properties, the Crosslinked PIS may be useful as an insulating layer of IGBT.

## Figures and Tables

**Table 1:** Molecular weights and PDI of PIS ^a^.

	$\bar{M}$_n_ (×10^4^ g/mol)	$\bar{M}$_w_ (×10^4^ g/mol)	PDI ^b^
PIS	1.9	4.4	2.4

^a^ Measured by GPC. ^b^ Polydispersity index ($\bar{M}$_w_/$\bar{M}$_n_).

**Table 2:** Crosslinking conditions of Allyl-PIS and state of the products.

Run No.	Allyl-PIS (wt%)	Crosslinker (wt%)	Catalyst (wt%)	Product ^a^
1	98.5	1	0.5	Viscous liquid
2	96.5	3	0.5	Semi-solid
3	94.5	5	0.5	Semi-solid (foamed)
4	92.5	7	0.5	Semi-solid (foamed)
5	98	1	1	Viscous liquid
6	96	3	1	Semi-solid
7	94	5	1	Semi-solid (foamed)
8	92	7	1	Semi-solid (foamed)
9	96.9	3	0.1	Viscous liquid
10	95	3	2	Semi-solid (foamed)

^a^ ‘Semi-solid’ means a non-foamed semi-solid, and ‘semi-solid (foamed)’ means a foamed semi-solid.

**Table 3:** Thermal properties of Crosslinked PIS.

	T_d_ (°C)		Thermal Diffusivity (m^2^/s)	Density (g/cm^3^)	Specific Heat Capacity (J/g·K)	Thermal Conductivity (W/m·K)
	T_5_^a^	T_10_^b^				
Crosslinked PIS	416	454	0.07	1.00	1.58	0.11

^a^ The temperature at which a sample exhibits 5 wt% decomposition in a nitrogen atmosphere. ^b^ The temperature at which a sample exhibits 10 wt% decomposition in a nitrogen atmosphere.

## Data Availability

Not applicable.

## Supplementary Materials

The following supporting information can be downloaded at: https://www.mdpi.com/article/10.3390/polym14245392/s1, Figure S1: Schematic diagram of a typical IGBT module; Figure S2: FT-IR spectrum of the commercial IGBT silicone gel; Figure S3: FT-IR spectrum of Allyl-PI; Figure S4: TGA curve of the commercial IGBT silicone gel; Figure S5: Transmittance of the commercial IGBT silicone gel; Figure S6: Storage modulus (G′) and loss modulus (G″) of the commercial IGBT silicone gel; Table S1: Remaining percent of Si-H groups; Table S2: Thermal properties of the commercial IGBT silicone gel.
