# Supplementary material for: Preparation of a Crosslinked Poly(imide-siloxane) for Application to Transistor Insulation
Source: Polymers (Basel). 2022 Dec 9;14(24):5392. doi: 10.3390/polym14245392 (PMC9782700; doi:10.3390/polym14245392)
Supplement: Supplementary file 1 [file polymers-14-05392-s001.zip › polymers-2061956-supplementary.pdf]

polymers

Supplementary Materials

Preparation of a Crosslinked Poly(imide-siloxane) for Application to Transistor Insulation

Hyeong-Joo Park, Ju-Young Choi, Seung-Won Jin, Seung-Hyun Lee, Yun-Je Choi, Dam-Bi Kim and Chan-Moon Chung *

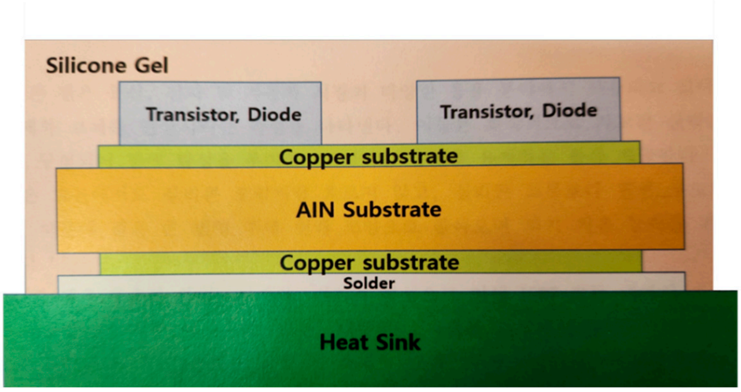

Figure S1. Schematic diagram of a typical IGBT module.

Characterization of IGBT silicone gel

The structure of the commercial IGBT silicone gel was identified by FT-IR spectroscopy. Absorption bands were observed at 1023 and 1098 cm^{-1} due to Si-O-Si stretching, and the absorption band of 1262 cm^{-1} is due to the symmetrical deformation of the $-CH_3$ group of $-Si(CH_3)_2-$ group. The absorption band of 803 cm^{-1} corresponds to Si-C group of $Si-CH_3$ vibration. It is considered that the silicone gel is a PDMS-based material because the IR spectrum of the gel is practically the same as that of PDMS.

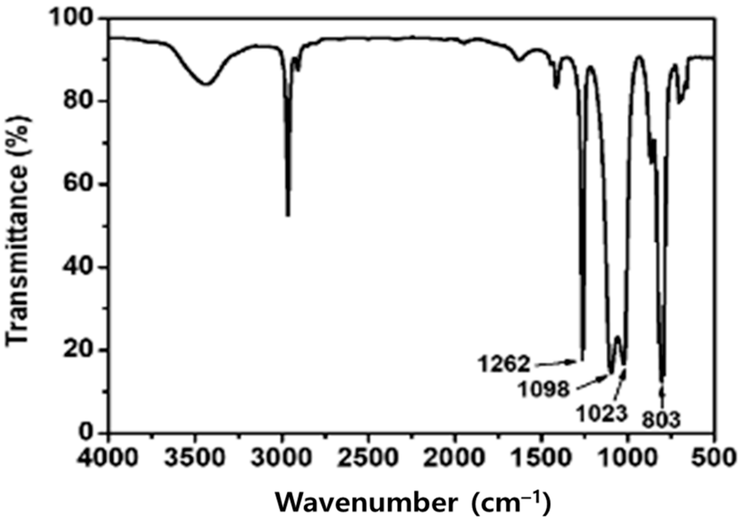

Figure S2. FT-IR spectrum of the commercial IGBT silicone gel.

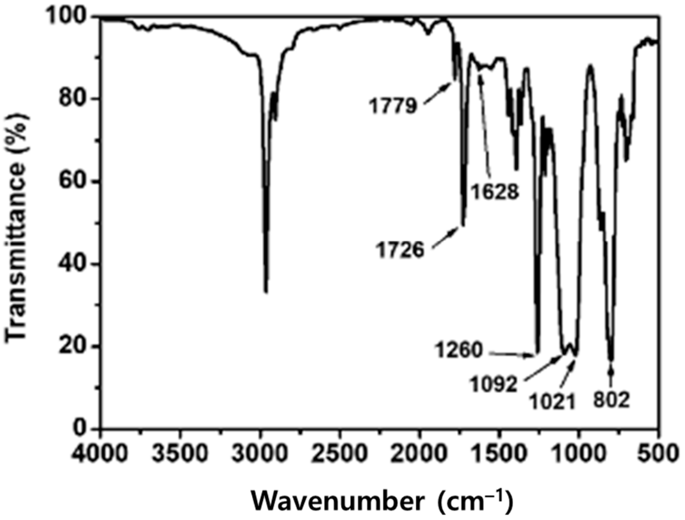

Figure S3. FT-IR spectrum of Allyl-PI.

Table S1. Remaining percent of Si-H groups.

Temperature (℃)	Residue (%)
Before Crosslinking	100
40	91
70	80
100	53
120	17
140	3

Table S2. Thermal properties of the commercial IGBT silicone gel.

	T_d (°C)		Thermal diffusivity (m^2/s)	Density (g/cm^3)	Specific heat capacity (J/g • K)	Thermal conductivity (W/m • K)
	T_5 [a]	T_{10} [b]				
IGBT silicone gel	378	422	0.12	1.00	1.67	0.19

[a] Temperature at which a sample exhibits 5 wt% decomposition in a nitrogen atmosphere; [b] Temperature at which a sample exhibits 10 wt% decomposition in a nitrogen atmosphere.

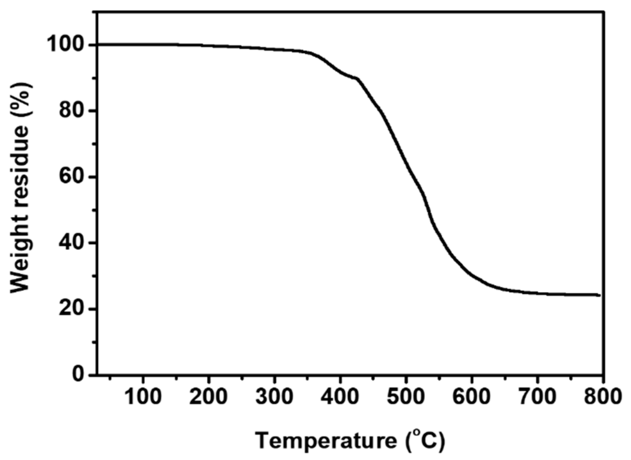

Figure S4. TGA curve of the commercial IGBT silicone gel.

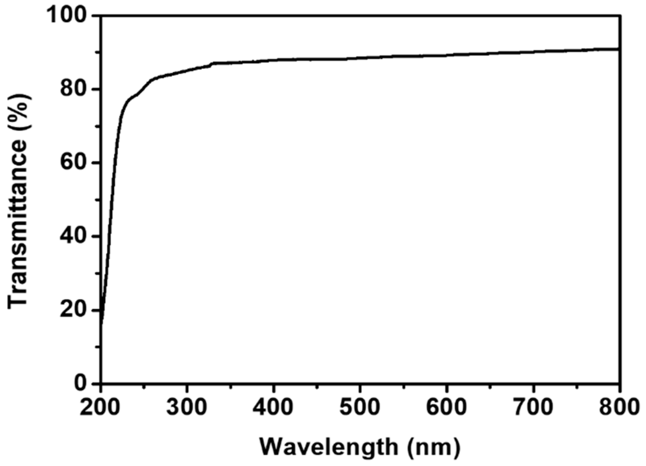

Figure S5. Transmittance of the commercial IGBT silicone gel.

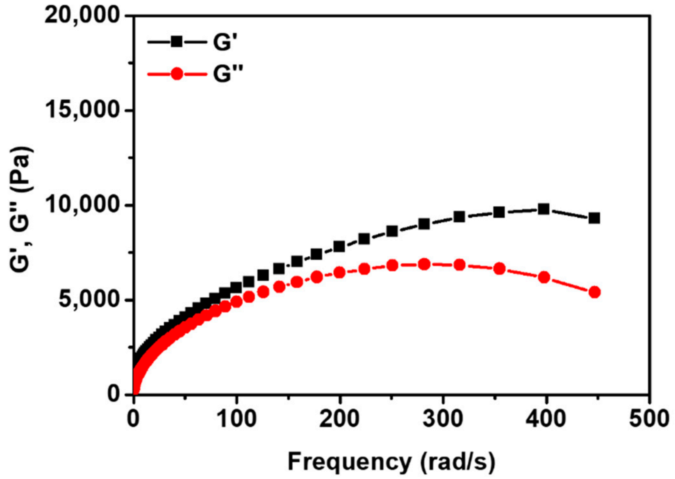

Figure S6. Storage modulus (G′) and loss modulus (G′′) of the commercial IGBT silicone gel.
